# Influence of Heat Treatment on the Microstructure and Mechanical Properties of Pure Copper Components Fabricated via Micro-Laser Powder Bed Fusion

**DOI:** 10.3390/ma17246270

**Published:** 2024-12-22

**Authors:** Shuo Qu, Liqiang Wang, Junhao Ding, Yang Lu, Xu Song

**Affiliations:** 1 Department of Mechanical and Automation Engineering, The Chinese University of Hong Kong, Hong Kong, China; qushuo@link.cuhk.edu.hk (S.Q.); jhding@link.cuhk.edu.hk (J.D.); 2 Department of Mechanical Engineering, City University of Hong Kong, Hong Kong, China; liqiawang2-c@my.cityu.edu.hk; 3 Department of Mechanical Engineering, The University of Hong Kong, Hong Kong, China; ylu1@hku.hk

**Keywords:** micro-laser powder bed fusion (μLPBF), microstructure, mechanical properties, heat treatment, pure copper

## Abstract

Pure copper (Cu) is widely used across numerous industries owing to its exceptional thermal and electrical conductivity. Additive manufacturing has facilitated the rapid and cost-effective prototyping of Cu components. Laser powder bed fusion (LPBF) has demonstrated the capability to produce intricate Cu components. However, LPBF-fabricated components exhibit anisotropic features, which stem from their inherent thermal gradients, resulting in properties that depend on the grain orientation. In the present study, pure Cu samples were fabricated via micro-laser powder bed fusion (μLPBF), resulting in improved mechanical properties, specifically, enhanced strength and ductility. The as-printed pure Cu sample exhibited thermal stability owing to its high-density grain boundaries and dislocations, enabling it to maintain relatively high levels of strength and ductility even when exposed to an elevated temperature of 300 °C. Furthermore, the heat treatment resulted in the disappearance of the initial microstructural characteristics, such as molten pool boundaries. As the heat-treatment temperature increased, the anisotropic yield strength decreased. Overall, the anisotropy of the properties of pure Cu components fabricated via μLPBF can be mitigated through heat-treatment-induced microstructural adjustments.

## 1. Introduction

The exceptional electrical and thermal conductivity of pure copper (Cu) material, which allows for heat transfer [1] and the manipulation of electromagnetic fields [2,3], make pure Cu extensively utilised across various industrial sectors. Additive manufacturing (AM) techniques allow for the utilisation of pure Cu components with intricate geometries and customisable properties [4,5]. The utilisation of powder bed fusion (PBF) technology is becoming prevalent owing to the growing demand for multi-functional components. PBF allows for the production of intricate and diverse structures at various scales while offering favourable thermal, electrical, and mechanical properties [6,7]. Electron beam powder bed fusion (EB-PBF) is renowned for achieving high densification in pure Cu components, overcoming the challenge of low laser energy absorptivity [8]. However, EB-PBF often results in an inferior surface quality and limited printing resolution, typically exceeding 500 μm [6,9,10]. Furthermore, this technique involves relatively low cooling rates and in situ annealing, contributing to the formation of extensive columnar grains, which reduces the mechanical strength [11].

Laser powder bed fusion (LPBF), another AM technique, offers distinct benefits over EB-PBF for fabricating components with intricate features and complex architectures [5]. However, producing pure Cu components with an excellent overall performance via LPBF is challenging, owing to the associated high thermal diffusion efficiency and infrared laser reflection [12]. Hence, the potential applications of LPBF are limited owing to the low relative density and inadequate strength and hardness of the fabricated components. In addition, the occurrence of grain coarsening and recrystallisation at elevated temperatures diminishes the ability of pure Cu to resist softening. The recrystallisation temperature of pure Cu is typically ~200 °C [13], and the underlying microstructure has a significant impact on this temperature. The presence of a self-stabilised dislocation network in as-printed (AP) pure Cu improves its mechanical performance through strengthening and hardening [14]. However, the strengthening effect of the initial microstructure can be weakened under heat treatment or elevated temperature conditions [15].

LPBF-fabricated components commonly display mechanical features that depend on the grain orientation. The grain morphology is associated with the building direction (BD). During fabrication, the heat flow aligns almost parallel to the BD, resulting in the growth of columnar crystals and thus the occurrence of anisotropic characteristics. This phenomenon introduces complexity into the AM design process, as the design process requires the consideration of material anisotropy. Numerous investigations have been conducted to analyse the anisotropic performance of LPBF-printed parts. One study analysed the anisotropic properties of as-built parts produced via EB-PBF [11]. The study revealed that the electrical conductivity and yield strength of these components were nearly isotropic. However, the strengthening process of the components was influenced by their texture. This dependency was ascribed to the significant grain growth that occurs in the in situ heat treatment process in EB-PBF. This study also demonstrates that EB-PBF can consistently produce components composed of pure Cu, exhibiting properties equivalent to those of pure Cu fabricated through more conventional processing methods, irrespective of the building orientation. Studies on metallic materials have attributed the observed anisotropy to the formation of elongated crystals due to epitaxial development [16,17,18]. Hence, an improvement in the mechanical property anisotropy through the recrystallisation of columnar grains following heat treatment has been observed in various metals processed using LPBF. Moreno et al. [19] demonstrated a significant reduction in the elastic anisotropy in samples of CM247LC, a nickel-based superalloy, after heat treatment above 1240 °C. This reduction in elastic anisotropy was attributed mostly to the disappearance of elongated grain microstructures in the samples, and only a minimal degree of anisotropy remained following the heat treatment. Chen et al. [20] reported that heat treatment had the potential to mitigate anisotropy in AlSi10Mg by promoting microstructural uniformity and redistributing internal stresses, which reveals the possibility of heat treatment to improve the components subjected to LPBF. Yang et al. [16] investigated the anisotropy of the alloy A357 in terms of its yield strength and ductility. The realisation of a more uniform microstructure through the heat treatment diminished the anisotropy. However, studies have not investigated the heat treatment of AP pure Cu samples fabricated via LPBF. Heat treatment significantly influences the grain evolution and anisotropic performance of pure Cu, particularly in terms of the high-temperature softening resistance properties. This could be helpful in microelectronic devices [17] and thermal management [18] applications, which involve diffusion welding at high temperatures.

In the present study, pure Cu components were manufactured via μLPBF, which involves the use of a fine laser beam, fine powders, and thin layer thickness. Our earlier studies [7,21] have shown that μLPBF can achieve AP pure Cu components with high relative density and superior performance. This is attributable to the method’s high energy density concentration, high laser power absorption of powders, and a large number of remelting cycles. The aim of this study is to elucidate the correlations between microstructural characteristics and the mechanical properties, specifically the tensile behaviour, of Cu components produced via LPBF. In addition, a range of heat treatment temperatures (500–900 °C) were considered to assess the correlation between the anisotropic properties and heat treatment parameters. Tensile studies conducted at elevated temperatures were also employed to evaluate the applicability of pure Cu in high-temperature environments.

## 2. Materials and Methods

### 2.1. Material and Process

**Figure 1:**
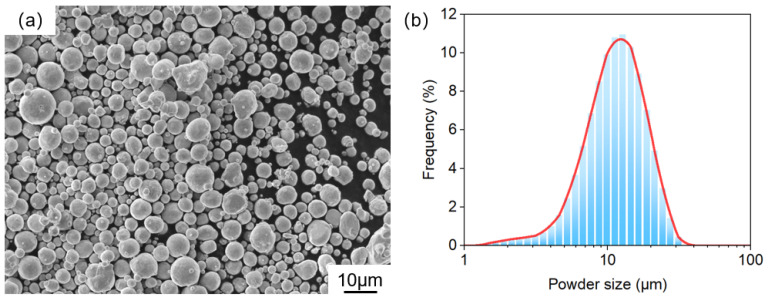
(**a**) Morphology of pure Cu powders, as observed via scanning electron microscopy; (**b**) distribution of powder size.

**Figure 2:**
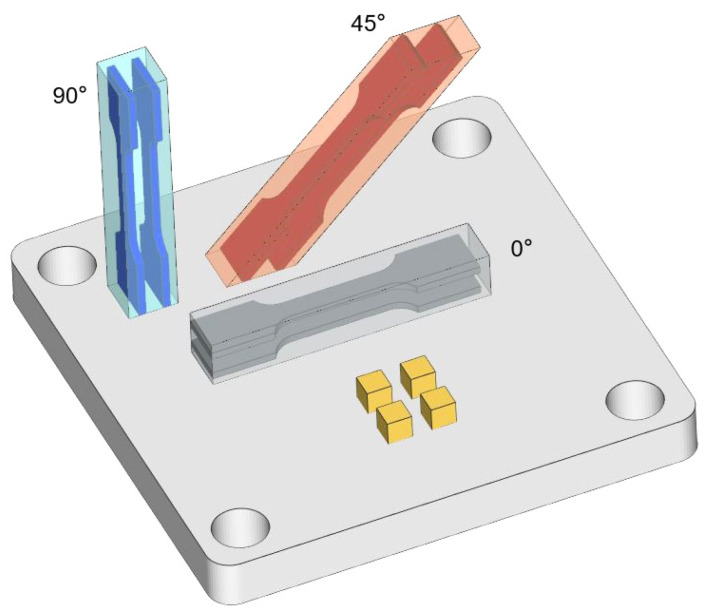
The illustrations of cube samples and tensile samples with different tensile directions (0/45/90°).

The samples were printed using an in-house μLPBF system, Han’s Laser M100µ [22]. The laser beam of the machine is 25 μm and layer thickness is 10 μm. The scan rotation angle is 67° between layers. The laser wavelength is 1070 nm and the protective atmosphere is nitrogen. The substrate material is pure Cu. Pure Cu powders of various particle sizes (0 to 25 μm) were obtained from Zhongyuan Advanced Materials Technologies (Changsha) Co. (Changsha, China). Figure 1 displays the shape and size conditions of the adopted fine powders. The process parameters can be found in a previous work [21]. Heat treatment was conducted using a furnace manufactured by Materials Research Furnace Inc. (Allenstown, NH, USA). To mitigate the oxidation of pure Cu, a nitrogen environment was adopted. The heating process was conducted at 50 °C/min, followed by the cooling of the furnace to reach 25 °C. The heat treatment parameters are chosen with a temperature of 500/700/900 °C and a holding time of 1 h. The AP samples exhibited relative densities exceeding 99.5%. Figure 2 shows the illustrations of cube samples and tensile samples with different tensile directions (0/45/90°). For convenience, the as-printed and 500/700/900 °C heat treatment samples with tensile directions of 0/45/90° are noted with AP/500/700/900-0/45/90°.

### 2.2. Microstructure Characterisation

The particle size distributions of the as-received powders were measured using a laser particle size analyser (Bettersize3000, Bettersize, Dandong, China). The relative density of samples was derived by the Archimedes method. The weight of pure Cu parts in the air and ethanol medium is measured by the analytical balance with an accuracy of 0.01 mg. The specimens were subjected to a cross-sectional view preparation process involving grinding, polishing and subsequent etching using a solution composed of 5 g of FeCl_3_, 85 mL of ethanol, and 15 mL of HCl. The as-received pure Cu particles were examined using a scanning electron microscope (JCM-6000PLUS, Jeol, Japan). Observations were also conducted using a transmission electron microscope (FEI Talos F200X, thermofisher, Waltham, MA, USA) operated at 200 kV. The morphology of AP cubes with dimensions of 5 mm × 5 mm × 5 mm was characterised via optical microscopy (OM) and electron backscatter diffraction (EBSD). Dog-bone samples with gauge dimensions of 15 mm × 4 mm × 1.5 mm were fabricated via wire-electrode cutting. Quasi-static uniaxial tensile experiments were conducted at both ambient and elevated temperatures using a Material Testing System (MTS) tension apparatus. The experiments were conducted under a consistent strain rate of 1 × 10^−3^ s^−1^. To obtain statistical data, a minimum of three tensile tests were performed. The initial powders and the fractures were examined using the JCM-6000PLUS scanning electron microscope. OM analysis was conducted using an RH-2000 high-resolution 3D microscope manufactured by Hirox, Japan. Grain structure and texture were investigated using the EDAX Velocity Plus EBSD system, EDAX, Mahwah NJ, USA. The data were collected at a step size of 0.5 μm. The EBSD data were analysed using MTEX 5.7.0, a Matlab toolbox [23]. Grain boundaries (GBs) were characterised according to their misorientation angle. Low-angle grain boundaries (LAGBs) are defined as GBs displaying misorientation angles ranging from 2° to 15°, while high-angle grain boundaries (HAGBs) are characterised by misorientation angles exceeding 15°. The presence of HAGBs affects the determination of grain size. In order to extract the grain size distribution and fractions, twin boundaries are excluded from the calculations in accordance with ASTM E2627-2013 [24].

## 3. Results

### 3.1. Pure Cu Sample Fabrication via μLPBF

**Figure 3:**
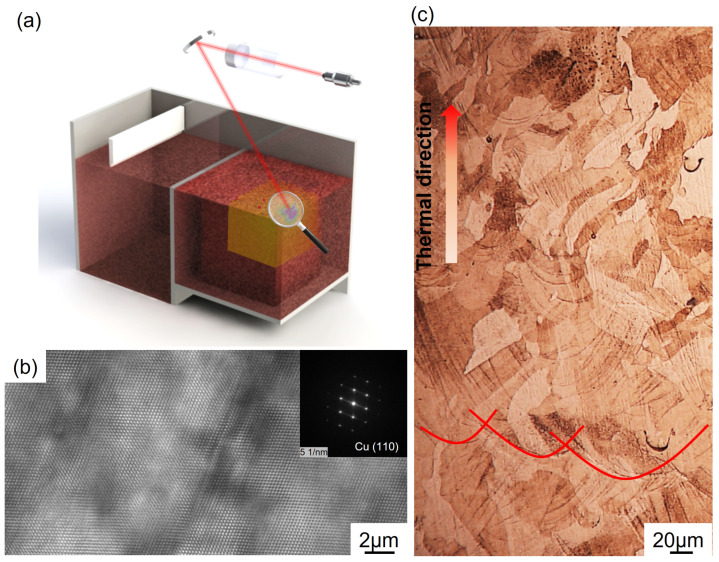
(**a**) μLPBF-based fabrication process; (**b**) Cu lattice along the (110) plane characterised via transmission electron microscopy; (**c**) OM-derived morphology of AP pure Cu sample along BD.

Figure 3 illustrates the fabrication process and the microstructural conditions of the AP Cu sample. Figure 3a shows the μLPBF process for pure Cu; the laser spot was narrowed owing to the presence of a large incident spot size [25]. The advantages of the μLPBF process for pure Cu have been presented in our previous studies [7,21]. The transmission electron microscopy-imaged morphology of the AP pure Cu sample (Figure 3b) showed that the lattice structure aligned with the (110) direction. Figure 3c shows the typical microstructure of the sample fabricated via the LPBF process. The molten pool introduced a significant thermal gradient during the process, which led to the occurrence of numerous elongated grains along the BD [26]. Multiple orientations of crystals were formed. Moreover, numerous elongated grains originated from the lower region of the molten pool and extended beyond its boundary. Additionally, numerous molten pool boundaries occurred on the side surface of the sample. Several studies have related these characteristics to anisotropic mechanical properties [7,27,28,29]. Therefore, microstructures of AP Cu subjected to different heat treatments were characterised, and mechanical evaluations were conducted to show the thermal stability and anisotropic properties of the AP pure Cu samples.

### 3.2. Microstructural Characterisation

**Figure 4:**
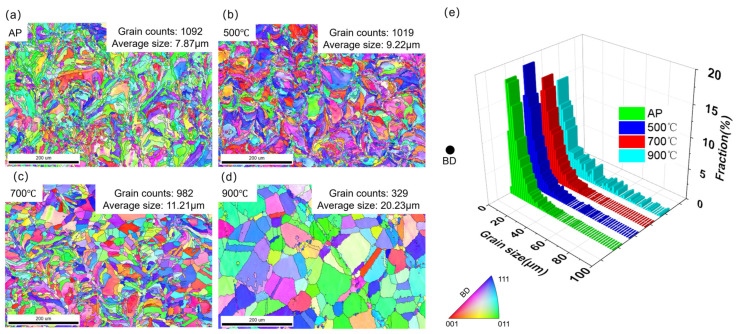
(**a**–**d**) EBSD grain orientation maps with the scan directions of the samples: (**a**) AP condition, (**b**) 500 °C condition; (**c**) 700 °C condition; (**d**) 900 °C condition; (**e**) grain size distribution of samples treated under the four conditions.

The horizontal and vertical cross-sections of the specimens were analysed via EBSD (Figure 4). The grain orientation maps are presented in accordance with the established convention, illustrating the BD for several conditions: the AP condition, the 500 °C treatment condition, the 700 °C treatment condition, and the 900 °C treatment condition. Figure 4d displays three surfaces, namely, the XY, YZ, and XZ planes, to visually represent the microstructures of the samples treated at 900 °C. Figure 4e displays the grain size distributions of four samples corresponding to the different conditions. The average grain size of each sample was determined from the respective EBSD grain map. The AP sample had an average grain size of ~8.02 μm. As the heat treatment temperature increased, the average grain size of the samples correspondingly increased. The sample treated at 900 °C exhibited a significantly larger grain size (19 μm) than that treated at 700 °C (10 μm). Regarding the grain morphology, the AP sample exhibited a distinct heterogeneous grain structure, characterised by the presence of both elongated grain zones and equiaxial grain zones. S-shaped grains were also formed during the μLPBF process (Figure 4a). Compared with the AP sample, the sample heat-treated for 1 h at 500 °C exhibited a lower relative abundance of fine grains (Figure 4b) and a higher abundance of coarse grains. However, the sample exhibited curved GBs and a molten pool. The sample heat-treated for 1 h at 700 °C exhibited larger grain sizes than that treated at 500 °C (Figure 4c). Over the treatment duration, the grains of the 700 °C-treated sample underwent equiaxialisation and growth, resulting in an increase in size, and the samples exhibited molten pool boundaries. The sample treated at 900 °C (Figure 4d) exhibited a grain size that was approximately twice as large as those of the other samples (Figure 4e). The elevated heat treatment resulted in the formation of annealing twins, which is attributable to a reduction in the stacking fault energy required for the formation of a twinned area [30]. In addition, the analysis of three surfaces exhibiting distinct cross-sections, as described by EBSD, revealed the presence of isotropic microstructures.

**Figure 5:**
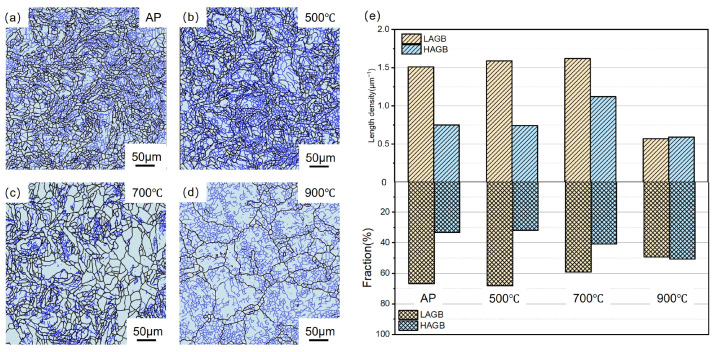
(**a**–**d**) GB maps of samples (blue: LAGB, black: HAGB): (**a**) AP condition, (**b**) 500 °C condition, (**c**) 700 °C condition, and (**d**) 900 °C condition; (**e**) length density of GBs and the HAGB and LAGB fractions in pure Cu samples under four different conditions.

Figure 5 illustrates the distribution of the GBs in the samples of four different conditions, which correspond to the specific areas depicted in Figure 4. In the figure, the black lines denote HAGBs, while the blue lines denote LAGBs. The AP sample exhibited a significant abundance of LAGBs. The length density was close to 1.5 μm^−1^, similar to those of the samples heat-treated at 500 °C and 700 °C. The sample heat-treated at 900 °C exhibited a significantly lower GB density than the other samples, presumably because the higher-temperature heat treatment resulted in greater LAGB and HAGB consumption than the other conditions (Figure 3a–c). Moreover, the HAGB fraction increased from 33% to 52%. The GB (LAGB and HAGB) density was significantly reduced to ~0.5 μm^−1^. Overall, the 900 °C-treated sample was significantly different from the samples heat-treated at other conditions.

**Figure 6:**
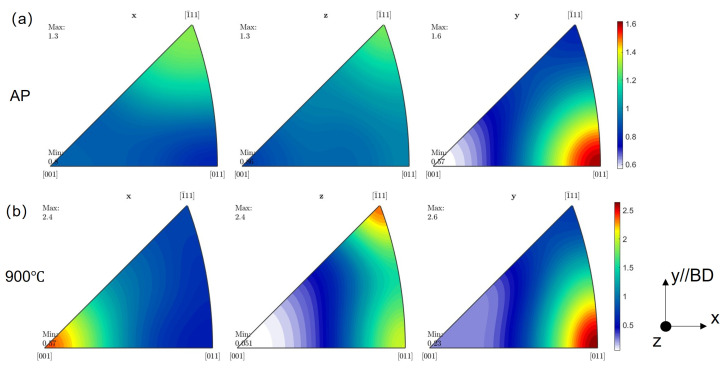
IPF map of samples: (**a**) AP condition; (**b**) 900 °C condition, along various directions.

**Figure 7:**
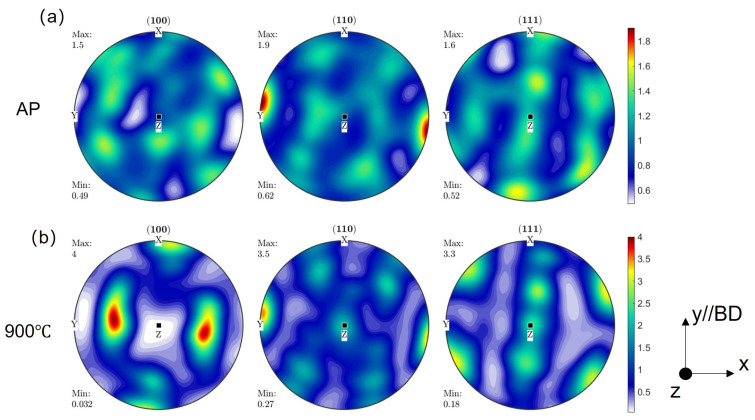
Orientation density function map of samples: (**a**) AP condition, (**b**) 900 °C condition, along various directions.

Figure 6 shows representative textures of the AP and 900 °C-treated samples. The AP sample featured a weak texture owing to the fine grains formed under the influence of different scan directions between layers. The inverse pole figure (IPF) of the AP sample (Figure 6) showed a relatively low intensity of texture <110>//BD. Following annealing for 1 h at 900 °C, the texture became stronger, exhibiting an increase in intensity from 1.6 to 2.6 along the scan direction. The orientation density function results showed a more concentrated distribution for the 900 °C-treated sample (Figure 7). The occurrence of three simultaneous processes—recovery, the development of recrystallised grains inside the deformed matrix, and the competition of grain growth within the completely recrystallised regions—is attributable to the heat treatment following the fabrication process. The relative distributions of these three processes underwent substantial changes over the heat treatment duration. The recrystallisation process in deformed regions and the competition of grain development within the clusters of recrystallised grains were driven by distinct factors; consequently, these processes showed varying rates of change [31]. Hence, grains exhibiting a distinct orientation might exhibit a preferential growth pattern. The texture observed at the last stage of primary recrystallisation was influenced by topological factors, such as the distribution of stored energy, the presence of nucleation sites, and the proximity of growing recrystallised grains. Additionally, the interaction between these factors played a significant role in shaping the recrystallisation texture [32]. The grain count number in each condition is shown in Figure 4, which is analysed according to ASTM E2627-2013. The TBs are defined as Σ3 coincidence site lattice boundaries with a misorientation angle and axis of 60° and <111> (face-centred cubic recrystallisation twin), and Σ9 coincidence site lattice boundaries with a misorientation angle and axis of 38.9° and <110> (face-centred cubic second recrystallisation twin) [33], respectively, which are excluded from the grain boundary analysis. The image boundary grains and twins are also excluded from the texture analysis.

### 3.3. Mechanical Properties at Room Temperature

**Figure 8:**
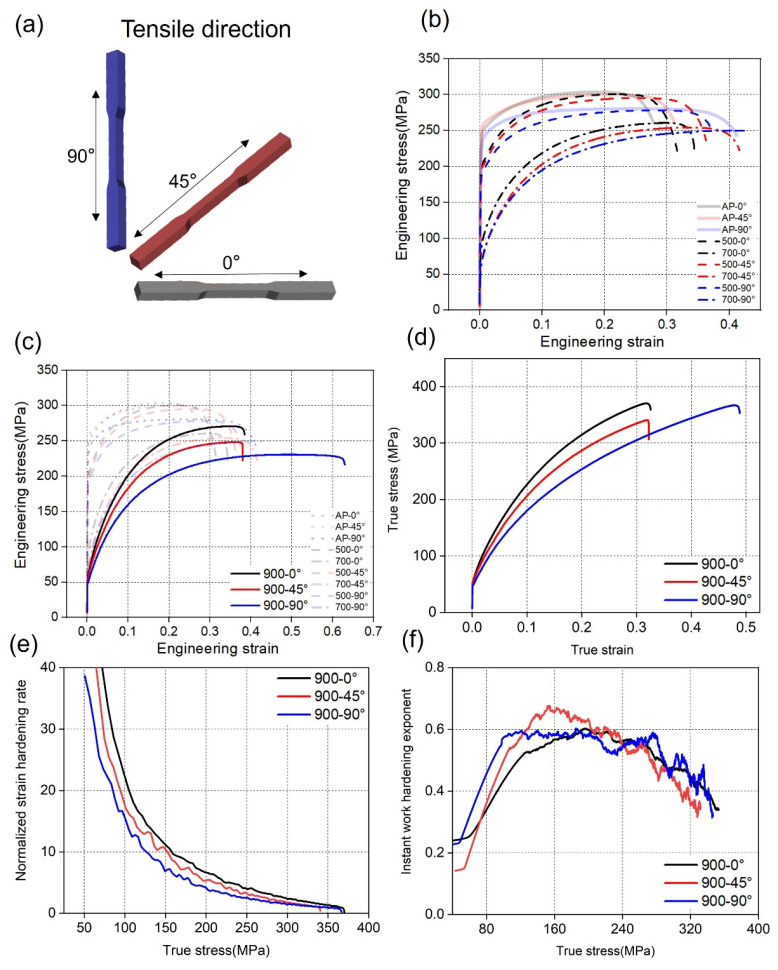
Mechanical properties of pure Cu samples. (**a**) Tensile test illustration for the 0°/45°/90° directions; (**b**) engineering stress–strain curves of the AP sample and the 500 °C- and 700 °C-treated samples; (**c**–**f**) properties of the 900 °C-treated sample in the 0°/45°/90° tensile directions; (**c**) engineering stress–strain curves; (**d**) true stress–strain curves; (**e**) normalised work-hardening rate; (**f**) instantaneous work-hardening exponents.

**Figure 9:**
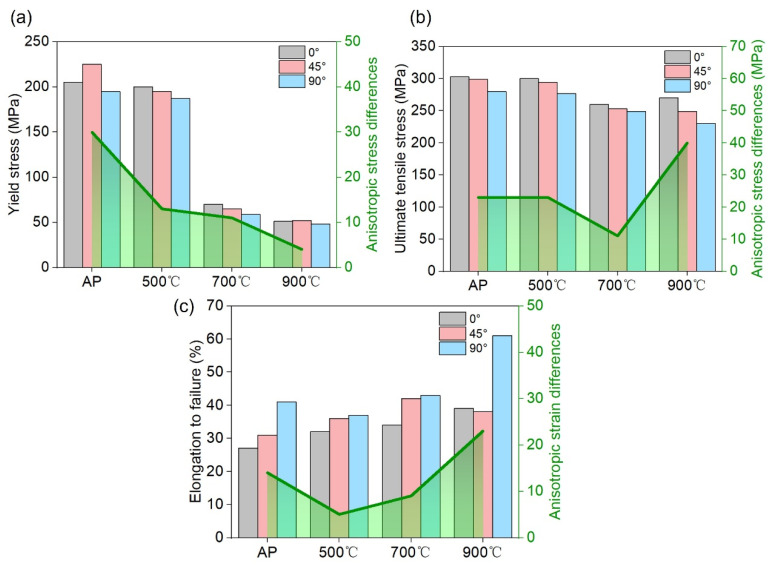
Mechanical properties and differences in anisotropy: (**a**) YS; (**b**) UTS; (**c**) elongation to failure.

Figure 8 depicts how the mechanical properties of the pure Cu samples varied depending on their respective conditions, as evidenced by the anisotropy observed in the stress–strain curves (Figure 8b). These curves were obtained from AP pure Cu tensile bars in different BDs (Figure 8a). The yield stress (YS), ultimate tensile stress (UTS) and elongation are shown in Figure 9. The distinctions between the values under various conditions are represented by green lines.

The 900 °C-treated samples exhibited a comparable YS (67 MPa) to a previously reported annealed pure Cu material [12]. The AP-90° specimens exhibited a greater elongation at failure (41.2%) than the AP-0° and AP-45° specimens. Samples AP-0° and AP-45° exhibited a higher YS than that of sample AP-90°. Among the 500 °C-treated samples, sample 500-0° exhibited the highest YS and UTS and the lowest elongation. The samples exhibited a weaker anisotropy of stress and strain in various directions than the AP samples. In contrast, the 500 °C-treated samples exhibited comparable mechanical properties to the AP samples. Among the 700 °C-treated samples, YS remarkably decreased from ~200 (500 °C-treated samples) to ~70 MPa. The elongation of the samples continued to increase with the increase in the heat treatment temperature. The rankings of stress and strain for different directions were the same as those for the 500 °C samples. In terms of anisotropy, the 700 °C-treated samples exhibited a lower stress difference and higher elongation difference in various directions compared with the 500 °C-treated samples. For the 900 °C condition, the elongation of sample 900-90° was over 60%, which led to a much higher difference in the UTS among various tensile directions compared with the samples treated under other conditions (Figure 9b). Moreover, sample 900-90° exhibited a nearly isotropic YS.

To further clarify the deformation behaviour, Figure 8e illustrates the progression of the normalised strain hardening rate (θ) [34]. This rate was determined using the following calculation method:

(1) $$\theta =\frac{1}{\sigma }\left(\frac{d\sigma }{d\varepsilon }\right)$$

where σ and ε represent the true stress and true strain, respectively. In the initial phase upon yielding, all of the samples exhibited a significant and fast reduction in θ.

The instantaneous work-hardening exponent n was calculated using the Hollomon equation:

(2) $$n=\frac{d\left(ln\sigma \right)}{d\left(ln\varepsilon \right)}$$

The 900 °C-treated samples (Figure 8f) exhibited a consistent pattern, characterised by a gradual rise in magnitude following the onset of yielding under similar stress conditions. During the post-necking stage, n reached its peak and then significantly declined. The YS of pure Cu was subject to various parameters, including the number of potential slip systems, the density of dislocations [14], and the geometric constraints placed on GBs [35].

The heat-treated samples often exhibited a higher ductility (ranging from 30% to 60%) than that of the AP sample (25–40%), attributable to the existence of grain refinement and dislocation networks in the heat-treated samples [36]. Fine-grained materials possess a high capacity to undergo deformation and effectively impede the formation of microcracks. Furthermore, molten pool boundaries are characterised by weak binding forces [37]. Some reported that failure occurred mostly at the molten pool boundaries, which led to the formation of weak areas and stress concentration [38], while the heat-treated samples exhibited a homogeneous distribution of GBs, resulting in even deformation and stress conduction [38]. Studies have revealed that LPBF-fabricated samples exhibited anisotropic tensile behaviour, with the 90° grain orientation demonstrating greater elongation than the 0° grain orientation [37,39,40]. Moreover, a previous study [7] verified that in an AP Cu material, the primary mechanism of deformation was dislocation slipping, rather than twinning. This suggests that the anisotropic deformation behaviour resulted from the presence of elongated crystals. An increase in the GB density resulted in a greater number of dislocations during the plastic deformation phase, thereby augmenting the work-hardening rate. Hence, the anisotropic ductility of the material is attributable to the variation in the GB density across distinct grain orientations, resulting in dissimilar mean-free pathways for dislocations. The 900 °C-treated samples exhibited comparable initial GB densities on each surface, resulting in an isotropic YS. The anisotropic ductility of the material is attributable to the presence of recrystallised grains with innate orientation preferences, leading to the occurrence of anisotropic dislocation slip behaviours. Moreover, the 900 °C-treated samples exhibited comparable work-hardening rates (Figure 8e,f), which indicates that the bulk materials exhibited an isotropic GB distribution, as shown in the grain orientation maps. The anisotropic elongation of the 900 °C-treated samples is attributable to the initial microcrack orientation and defect shapes, which could not be modified by the heat treatment. It causes fractures to extend along the scan direction, lowering the ductility.

**Figure 10:**
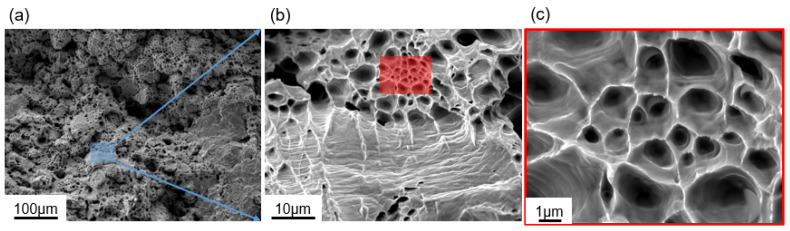
(**a**–**c**) Fracture morphologies of the 900 °C-treated sample, as observed via scanning electron microscopy at different magnifications.

Figure 10 depicts the fracture morphology of the pure Cu samples subjected to heat treatment. All of the samples, irrespective of the treatment settings, exhibited necking. The characteristic fracture morphology, as observed via scanning electron microscopy (Figure 10a), revealed the presence of numerous dimples, corresponding to ductile fracture [41]. Figure 10b is the enlarged view of Figure 10a, showing the details of the fracture morphology. The 900 °C-treated samples (Figure 10c) exhibited grain slip with much larger dimples (~1 μm) than the AP Cu samples [41,42], consistent with the analysis of the mechanical results shown in Figure 8 and Figure 9. Ductile fracture was often the predominant fracture mode in the annealed samples.

### 3.4. Mechanical Properties at Elevated Temperatures

**Figure 11:**
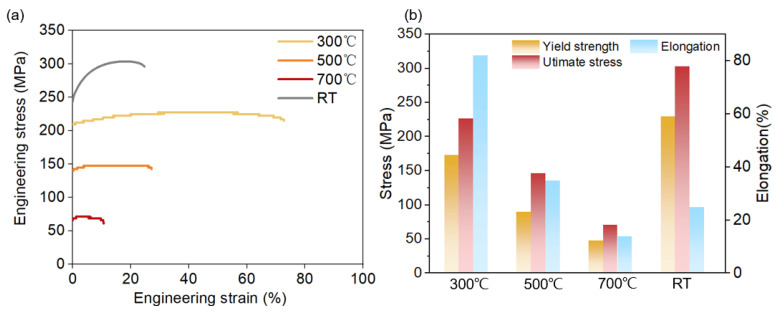
(**a**) Engineering stress–strain curve of AP pure Cu at high temperatures of 300 °C, 500 °C and 700 °C; (**b**) YS, UTS and elongation values under different conditions.

The mechanical properties of the AP Cu sample exposed to elevated temperatures were further evaluated to evaluate its heat stability. Figure 11 illustrates the mechanical behaviour of the AP pure Cu sample in a high-temperature environment. Testing temperatures of 300 °C, 500 °C and 700 °C were considered. The YS remained over 150 MPa when the testing temperature was 300 °C, while the elongation of the sample exposed to 300 °C was ~80%. At a temperature of 500 °C, the sample exhibited an elongation below 30%, while the YS was ~90 MPa. At 700 °C, the sample exhibited an elongation below 15%, while the YS was ~50 MPa.

Generally, the metal became more ductile and lost strength at high temperatures. As the temperature increased, both the intragranular strength and intercrystalline strength [43] decreased, which was beneficial for coordinated deformation. This excellent plasticity is attributable to the high-temperature effect and the fine-grain microstructure [44]. The pure Cu sample fabricated via LPBF also showed a high-temperature embrittlement effect. The ductility maintained a constant value at 300 °C throughout the tensile test but started to significantly decline at 500 °C. Accordingly, the critical temperature for the high-temperature embrittlement of pure Cu was 500 °C. The high-density GBs and dislocations led to better thermal stability compared with the case of commercial pure Cu (T2) [45].

## 4. Conclusions

This study examined the influence of heat treatment on the microstructure and mechanical properties of pure Cu. Compared with the conventional pure Cu parts, the pure Cu fabricated via μLPBF demonstrated a superior thermal stability, attributable to its inherently high GB density. The key findings of this study are summarised as follows:

1. As the heat treatment temperature increased, the average grain size increased from ~8 to 18 μm. The molten pool boundaries disappeared under 900 °C heat treatment, and the GB (LAGB and HAGB) density was significantly reduced to ~0.5 μm^−1^.
2. The anisotropy of the YS decreased as the heat treatment temperature increased from 300 °C to 900 °C, with the YS difference between samples decreasing from approximately ~30 MPa to ~4 MPa. Similarly, the anisotropy of elongation was weak under the 300 °C heat treatment but became more pronounced at 900 °C owing to the recrystallisation texture.
3. The high-temperature tensile performance results suggested guidelines for pure Cu under high-temperature service conditions. The sample exhibited significantly higher elongation at 300 °C (80%) than that at room temperature (25%), owing to the presence of a fine-grained microstructure, and a lower YS (~170 vs. ~220 MPa). At higher temperatures, the elongation and strength deteriorated significantly owing to elevated-temperature ductility loss (embrittlement).

## Data Availability

The original contributions presented in the study are included in the article, further inquiries can be directed to the corresponding author.
